# Accuracy of a pre-trained sentiment analysis (SA) classification model on tweets related to emergency response and early recovery assessment: the case of 2019 Albanian earthquake

**DOI:** 10.1007/s11069-022-05307-w

**Published:** 2022-03-23

**Authors:** Diana Contreras, Sean Wilkinson, Evangeline Alterman, Javier Hervás

**Affiliations:** 1 Centre for Resilience and Environmental Change (CHANGING), School of Earth and Environmental Sciences, College of Physical Sciences and Engineering, Cardiff University, Main Building, Park Place, Cardiff, CF10 3AT UK; 2 Learning from Earthquakes (LfE), School of Engineering, Faculty of Science, Agriculture and Engineering, Newcastle University, 2nd Floor Drummond Building, Newcastle, NE1 7RU UK; 3 Department Civil Engineering, Faculty Engineering, Auckland University, Private Bag 92019, Auckland, 1142 New Zealand; 4 77 Landmark Place, Churchill Way, Cardiff, CF10 2HS UK

**Keywords:** Earthquakes, Social media (SM), Twitter, Sentiment analysis (SA), Machine learning algorithm, Accuracy (ACC)

## Abstract

Traditionally, earthquake impact assessments have been made via fieldwork by non-governmental organisations (NGO's) sponsored data collection; however, this approach is time-consuming, expensive and often limited. Recently, social media (SM) has become a valuable tool for quickly collecting large amounts of first-hand data after a disaster and shows great potential for decision-making. Nevertheless, extracting meaningful information from SM is an ongoing area of research. This paper tests the accuracy of the pre-trained sentiment analysis (SA) model developed by the no-code machine learning platform MonkeyLearn using the text data related to the emergency response and early recovery phase of the three major earthquakes that struck Albania on the 26th November 2019. These events caused 51 deaths, 3000 injuries and extensive damage. We obtained 695 tweets with the hashtags: #Albania #AlbanianEarthquake, and #albanianearthquake from the 26th November 2019 to the 3rd February 2020. We used these data to test the accuracy of the pre-trained SA classification model developed by MonkeyLearn to identify polarity in text data. This test explores the feasibility to automate the classification process to extract meaningful information from text data from SM in real-time in the future. We tested the no-code machine learning platform's performance using a confusion matrix. We obtained an overall accuracy (ACC) of 63% and a misclassification rate of 37%. We conclude that the ACC of the unsupervised classification is sufficient for a preliminary assessment, but further research is needed to determine if the accuracy is improved by customising the training model of the machine learning platform.

## Introduction

After an earthquake, it is necessary to understand its impact to provide relief and improved mitigation strategies. The post-disaster period is divided into four post-disaster phases according to the United Nations Development Programme (UNDP): emergency phase, early recovery, recovery, and development phase. During the relief or emergency response phase, the priority is to save lives by deploying search and rescue (SAR) task forces (Alexander 2006; Kates and Pijawka 1977), surveying buildings to determine their degree of damage (Contreras et al. 2021) and to estimate the need for temporary shelters. The early recovery phase aims to start the return of the community to everyday life by removing debris, rehabilitating roads, demolishing damaged buildings (Alexander 2006; Brown et al. 2010), and start closing temporary shelters (Contreras 2022). The main objective during the recovery phase is for the disaster area to return to normality (Alexander 2006) through the continuous implementation of the recovery plan. The development phase focuses on improving upon the state that existed before the event (Chang 2009) implementing the lessons learnt from the earthquake (Contreras 2022).

Traditionally, impacts have been assessed via fieldwork missions by non-governmental organisations (NGO's) sponsored data collection; however, this approach is time-consuming, expensive and often limited (Contreras et al. 2021g). Recently, social media (SM) has become a valuable tool for quickly collecting large amounts of first-hand data after a disaster, as was demonstrated during the remote missions for the 2020 Zagreb earthquake (Contreras et al. 2021a; So et al. 2020a, b) and the 2020 Aegean earthquake (Aktas et al. 2021). These earthquakes occurred during the lockdown imposed by governments worldwide to reduce the population's exposure to COVID-19. This measure made it infeasible to conduct on-site fieldwork by non-locals for both cases. Therefore, remote missions were conducted with the support of local people who were inexperienced in data collection after an earthquake but supervised by experts in The United Kingdom (UK) who had previously conducted these types of surveys. Text and image data from SM showed great potential to support decision-making relevant to this event. These data were used to select the inspection areas undertaken by local students for the earthquake reconnaissance missions mentioned before. However, converting any collected text or image data into meaningful information that can support relief and recovery efforts is an ongoing area of research. This paper tests the accuracy of unsupervised classification of text data related to the emergency response and early recovery phase of the three major earthquakes that struck Albania on the 26th November 2019. This test explores the feasibility of automating the classification process to extract meaningful information from text data from SM after earthquakes using a no-code machine learning platform accessible for emergency response practitioners without coding skills to make data-driven decisions.

**Fig. 1:**
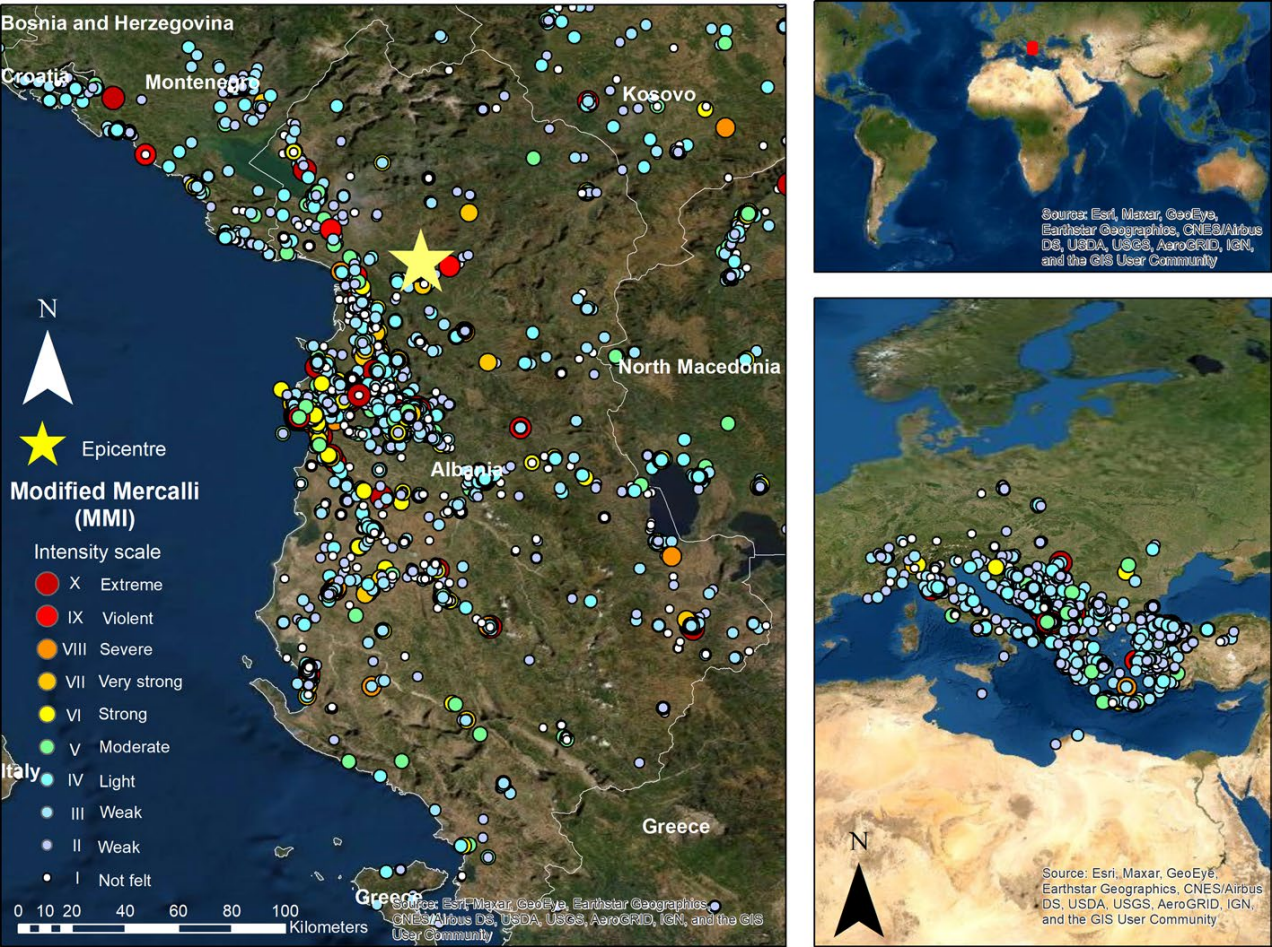
Epicentre and intensity reports after the Albanian earthquake in 2019.* Data Source*: Euro-Mediterranean Seismological Centre (EMSC)

The 2019 earthquake series in Albania started with an M_W_ 5.6 earthquake at 15:15 CET on the 21st September (Andonov et al. 2020a, b). However, the earthquake investigated in this paper had a moment magnitude M_W_ 6.4, a focal depth of 20 km and struck Albania's northwest region at 03:54 central European time (CET) (Bossu et al. 2020; Freddi et al. 2021). The epicentre was 16 km west-southwest of the town of Mamurras in Kurbin municipality (41.511°N 19.522°E). It was the strongest earthquake in Albania for the last 40 years, causing damages in the municipalities of Durrës, Lezhë, Tiranë (Bossu et al. 2020; IFRC 2021), Krujë, Shijak, Kamëz, Kavajë, and Kurbin (Freddi et al. 2021), mainly in the city of Durrës, the village of Kodër-Thumanë and the town of Laç. This earthquake happened two days before the celebration of the Independence of Albania on the 28th November. The second shock had an M_W_ 5.1, and the third and largest aftershock had M_W_ 5.4 and occurred at 07:10 CET (IFRC 2021) on the same day. The seismic activity continued until the beginning of January with a regular M_W_ 4 (Andonov et al. 2020a, b). The location of the epicentre and intensity reports in the Modified Mercalli Intensity Scale (MMI) of the first earthquake on the 26th are depicted in Fig. 1.

**Fig. 2:**
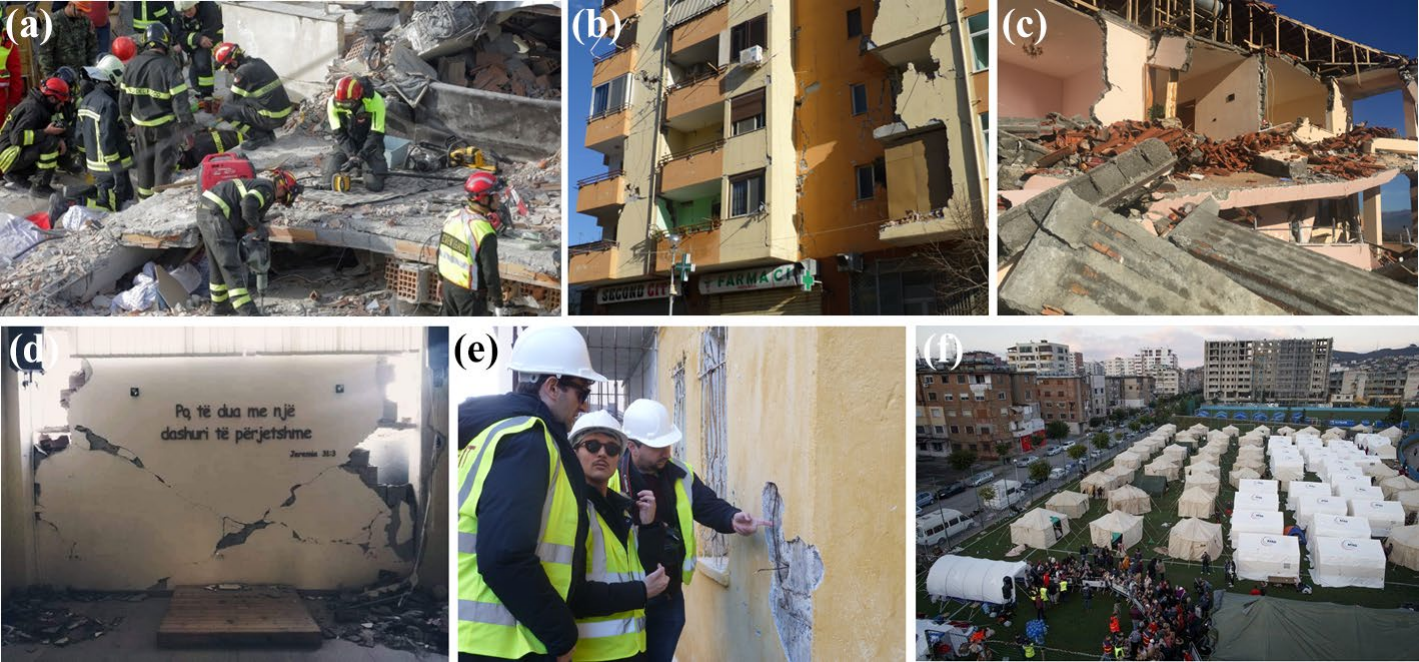
Damages after the earthquakes in Albania on the 26th November 2019: **a** SAR operations after the earthquake; **b** Walls broken due to the earthquake; **c** Collapsed house.* Source*: Andonov et al. (2020a, b). **d** Broken wall due to the earthquake.* Source*: Watchman (2019) **e** The earthquake engineering field investigation team (EEFIT) checking structural damages during the earthquake reconnaissance mission.* Source*: EEFIT. f Distribution of humanitarian aid in a temporary shelter.* Source*: ΑΠΕ-ΜΠΕ (2019)

The earthquake caused 51 deaths (Bossu et al. 2020), 47 due to the collapse of nine buildings and between 600 (Bossu et al. 2020) and 3000 injured (Andonov et al. 2020a, b). One thousand two hundred people were evacuated in Thumanë, Tiranë, Durrës, Krujë, and Lezhë (Reliefweb 2019). Emergency workers, soldiers and police searched through debris of buildings, where people were trapped. Most fatalities were located in Durrës and Thumanë, 40 kms northwest of Tiranë, Albania's capital. Albania was not adequately prepared to respond to the emergency generated by this earthquake. Therefore, a call for international support was made by the government. The European Commission (EC) deployed search and rescue (SAR) teams from Italy, Greece and Romania. Teams from Kosovo and Montenegro also arrived at the area to support SAR operations, as depicted in Fig. 2a. (BBC 2019). Consolidated reports indicated 11,490 housing units categorised as either fully destroyed, demolished, or in need of complete rebuild (see Fig. 2b and c). Additionally, 83,745 housing units were partially or slightly damaged (see Fig. 2d and e). Seventeen thousand people were displaced and living in temporary accommodation, first in camps (see Fig. 2f), in tents (not appropriate for winter), or rented accommodation (IFRC 2021) and hotels (Andonov et al. 2020a, b). The post-disaster need assessment (PDNA) reported that the estimated total effect of the disaster was €985 million (€ 844 million, representing the value of destroyed physical assets and €141 million, referred to as losses Andonov et al. 2020a, b).

Modern procedures for earthquake intensity assessment use two primary sources: physical sensors (seismographs and accelerometers) and social sensors (witness reports) (Kropivnitskaya et al. 2017a, b), such as the LastQuake app (Bossu et al. 2018b, a; Bossu et al. 2020). These social sensors have the potential to speed up the procedures for intensity calculations (Kropivnitskaya et al. 2017a, b). Instead, Twitter is a platform to get situational awareness (Eligüzel et al. 2020) during the emergency or relief phase (Contreras 2016) after a disaster. A correlation between the number of tweets and the intensity of an earthquake was observed for the first time in 2010 during the Tohoku earthquake. Researchers observed a high correlation between the number of tweets and the earthquake's intensity in some locations (Doan et al. 2012). The correlation between the number of tweets and Mercalli intensity was demonstrated in the earthquakes of Napa (California) (Kropivnitskaya et al. 2017a, b), Japan, and Chile (Kropivnitskaya et al. 2017a, b). Twitter encourages a fast generation of data in the minutes to hours and days following an event by exchanging information and active discussion between scientists themselves and stakeholders (Lacassin et al. 2020). Earthquake detection and SM analysis is a current active field of study (Earle et al. 2010; Sakaki et al. 2010; Burks et al. 2014; Contreras et al. 2021a). Social media provides opportunities for citizens to engage in emergency management by disseminating information to the public and providing them with access to it (Simon et al. 2015). Social media is also helpful to mobilise the population and provide them with the most up-to-date information, which might not be available through alternative official channels (Lerman and Ghosh 2010). Experiments using Twitter confirmed that people act as sensors providing comparable results promptly and complementing other data sources, enhancing situational awareness and improving the understanding and response to those events (Crooks et al. 2013). Neppalli et al. (2017) performed a SA related to Hurricane Sandy using the SentiStrength algorithm to classify the tweets. The authors found that polarity on tweets changes according to the user's distance to the event. Ragini et al. (2018) used SA with a lexicon-based approach to automatically determine on tweets the needs of people affected by the India-Pakistan floods in September 2014. Singh et al. (2019) used ‘R’ package 'twitteR' to analyse Twitter data related to the Las Vegas shooting in October 2017 with the help of 'tm' package in R to infer the spatiotemporal sentiments of the users. In the last three cases, all authors have used tools that require knowledge of algorithms and/or coding skills, and in none of the case studies, the phenomenon to tweet about were an earthquake. Only, Eligüzel et al (2020) used the ‘earthquake’ keyword to determine the location of Twitter users to demonstrate the importance of the location in disaster management by utilising geotagged tweets. However, these authors evaluated the tweets employing Latent Dirichlet Allocation (LDA) topic model and SA through 10 machine learning algorithms (i.e. Multinomial and Gaussian Naive Bayes, Support Vector Machine (SVM), Decision Tree, Random Forest, Extra Trees, Neural Network, k Nearest Neighbor (kNN), Stochastic, Gradient Descent (SGD), and Adaptive Boosting (AdaBoost) classifications, which are not familiar to emergency response practitioners.

Earthquake-related studies propose to use data mining and natural language processing (NLP) for damage detection and assessment of earthquakes (Avvenuti et al. 2014). These research studies suggest applying classifier methods for earthquake detection (Sakaki et al. 2010; Robinson et al. 2013). They propose using probabilistic spatiotemporal models for reporting earthquake-related events (Sakaki et al. 2013). Other studies use qualitative approaches to analyse population behaviour after an earthquake (Miyabe et al. 2012), applying a keyword level analysis to track social attitudes (Doan et al. 2011) and analysing the dynamic of rumour mills in tweets (Oh et al. 2010; Karami et al. 2020). The extraction of sentiments mainly from text data during a disaster contributes to a vital situational awareness of the disaster zone dynamics. Wu and Cui (2018) used sentiment analysis (SA) on tweets related to Hurricane Sandy to measure each tweet's emotion or mood and classified it as positive, negative, or neutral. They confirmed that the severity of damage in the area was correlated with disaster-related activity, e.g. distress calls, SAR operations, humanitarian aid distribution, and cleaning afterwards. Contreras et al. (2021a, d, h) performed the assessment of post-disaster recovery in L’Aquila, Italy, during the 10th anniversary using SA. According to Ragini et al. (2018), the sentiment of tweets during and after a disaster is an indicator of the success or failure of the emergency response. Sentiment analysis, also called opinion mining, is an NLP method used to automatically analyse (Hausmann et al. 2020) the text data (Garreta et al. 2019) through computational treatment, assessing sentiments (Taboada et al. 2011), emotions, opinions, attitudes, and subjectivity about a specific topic or towards an entity (Medhat et al. 2014; Zucco et al. 2020). To analyse the text's emotional load, it is essential to understand its meaning (Gurman and Ellenberger 2015; Ragini et al. 2018). Sentiment analysis identifies the sentiments in the text and classifies their polarity into positive, negative or neutral (Ragini et al. 2018). This classification can be performed at three levels of granularity: document-level, sentence-level, and sub-sentence level (MonkeyLearn 2020a, b, c) or aspect-level (opinion and target) (Liu 2015). There are sentiment words or opinion words, which are words in a language that indicates desirable of undesirable states. Examples of positive sentiment words are beautiful, good and great. Examples of negative words are dreadful, bad and awful (Liu 2015). Two techniques are used for SA: machine learning and lexicon-based (Khan and Thakare 2015). The machine learning approach can be supervised or unsupervised.

The main aim of using SM is to extract meaningful information from image and text data in pictures and posts in SM platforms to support data-driven decisions in earthquake reconnaissance and emergency response. These data support the preparation of PDNA, the assessment of emergency response and post-disaster recovery processes (Contreras et al. 2021a, b, c, d, e, f, g) and improve preparedness in the future. However, it is necessary to identify the right approach to extract the information in the shortest time possible from the large datasets collected from SM that enables accurate situational awareness of the event and improves the ongoing and future emergency response operations after an earthquake. To aid in this objective, we test the accuracy of the pre-trained model of SA classification developed by MonkeyLearn, a machine learning platform for text analysis (Wolff 2020). This pre-trained model performs an unsupervised classification of text data, in this case, data related to the emergency response and early recovery phase after the earthquakes in Albania. Later we compare it to the outcomes of a supervised classification from experts. We aim to check the feasibility of automating the SA classification process with an acceptable accuracy rate. This tool developed by MonkeyLearn was selected because it does not require coding skills, making it accessible to any population group such as emergency response practitioners. In the project framework: Learning from Earthquakes UK (LfE-UK), a joint project undertaken by Newcastle University, the University College of London (UCL) and Cambridge University 695 Tweets with the hashtags: #Albania, #AlbanianEarthquake and #albanianearthquake from the 26th November 2019 to the 3rd February 2020 were collected. These tweets are used in this research to test the accuracy of a pre-trained model of SA classification.

This paper is divided into five sections. The introductory section presents the case study area, describes the emergency after the earthquake, defines the purpose of the research and includes a literature review. The materials and method section details the methodology applied, and the results sections include the outcomes of the applied methodology. The discussion section interprets the general classification results and explains the results obtained, including samples of the tweets classified. The conclusion section recalls the purpose of the research regarding the methodology applied and results, a summary of the rules defined by authors for the polarity classification, and findings. It ends with exploring opportunities for a further test of pre-trained algorithm performance and includes recommendations for additional research.

## Materials and methods

In the project framework: Learning from Earthquakes UK (LfE-UK), 675 Tweets with the hashtags: #Albania, #AlbanianEarthquake and #albanianearthquake from the 26th November 2019 to the 3rd February 2020 were collected by a third-party vendor. The SM department of Newcastle University provided us with 1001 tweets with the hashtag #Albania collected from the 31st January to the 2nd February 2020. We obtained the text data from both sources in a report in excel. We integrated these two datasets and discarded those unrelated tweets, resulting in a database of 695 tweets. Tweets related to this event were written in English, Estonian, modern Greek, Icelandic, French, German, Catalan, Spanish, Norwegian, and other languages that could not be identified. We processed the data, which involved eliminating Twitter handles and hyperlinks, translating the tweets to English, and removing repeated tweets from the database, leaving us with a dataset of 255 original tweets (38% of the original dataset).

This dataset of original tweets was used to test the accuracy of the classification algorithm developed by MonkeyLearn. We used the MonkeyLearn algorithm to identify polarity in the text data collected from Twitter and related to the earthquake's emergency response and early recovery phase in Albania. This algorithm-based classification, also known as unsupervised or automatic classification, will be referred to as 'predicted' for the rest of this paper. The main result of running the tweets through the algorithm is classifying the tweets into a specific polarity with a degree of confidence (note: the degree of confidence is a "black-box" metric provided by MonkeyLearn and we include it in this work, so we can not only compare the accuracy of the algorithm, but also the confidence the algorithm has in its classification). At the same time, through combining the experience in emergency management of the first author and monitoring post-disaster recovery processes from the first two authors, the rules to classify the polarity of the tweets were defined (Alterman 2020) and are referred to as a 'supervised' classification. Tweets that mention actions that contribute to achieving the main objectives of the emergency response and the early recovery phases (Contreras 2016) after the earthquake will be considered positive. In contrast, tweets that report events and actions that reduce the possibility of accomplishing those objectives will be classified into a negative polarity. Tweets that report the characteristics of the event without mentioning any particular action will be classified as neutral. The complete and specific rules set to define the polarity of tweets related to the earthquake in Albania in 2019 are listed in Table 1.

**Table 1:** Rule-set for polarity classification of tweets during the emergency and early recovery phases after earthquakes

Polarity	Rules
Positive (Pt)	Donations and fundraising activities Encouraging messages Humanitarian aid supplies International support to the government of the affected country Prominent people visiting the affected area Search and rescue (SAR) activities Solidarity messages
Negative (Ng)	Calls from the government of the affected area for international support Comments about aftershocks Complaints about bad construction practices Complaints about previous damage assessment Criticism to the emergency management Damages in buildings Expressions of anxiety and fear Injuries and casualties Xenophobia messages
Neutral (Nt)	Seismic information Request of information Technical specifications of humanitarian items General information about the case study area (e.g. number of inhabitants)

**Fig. 3:**
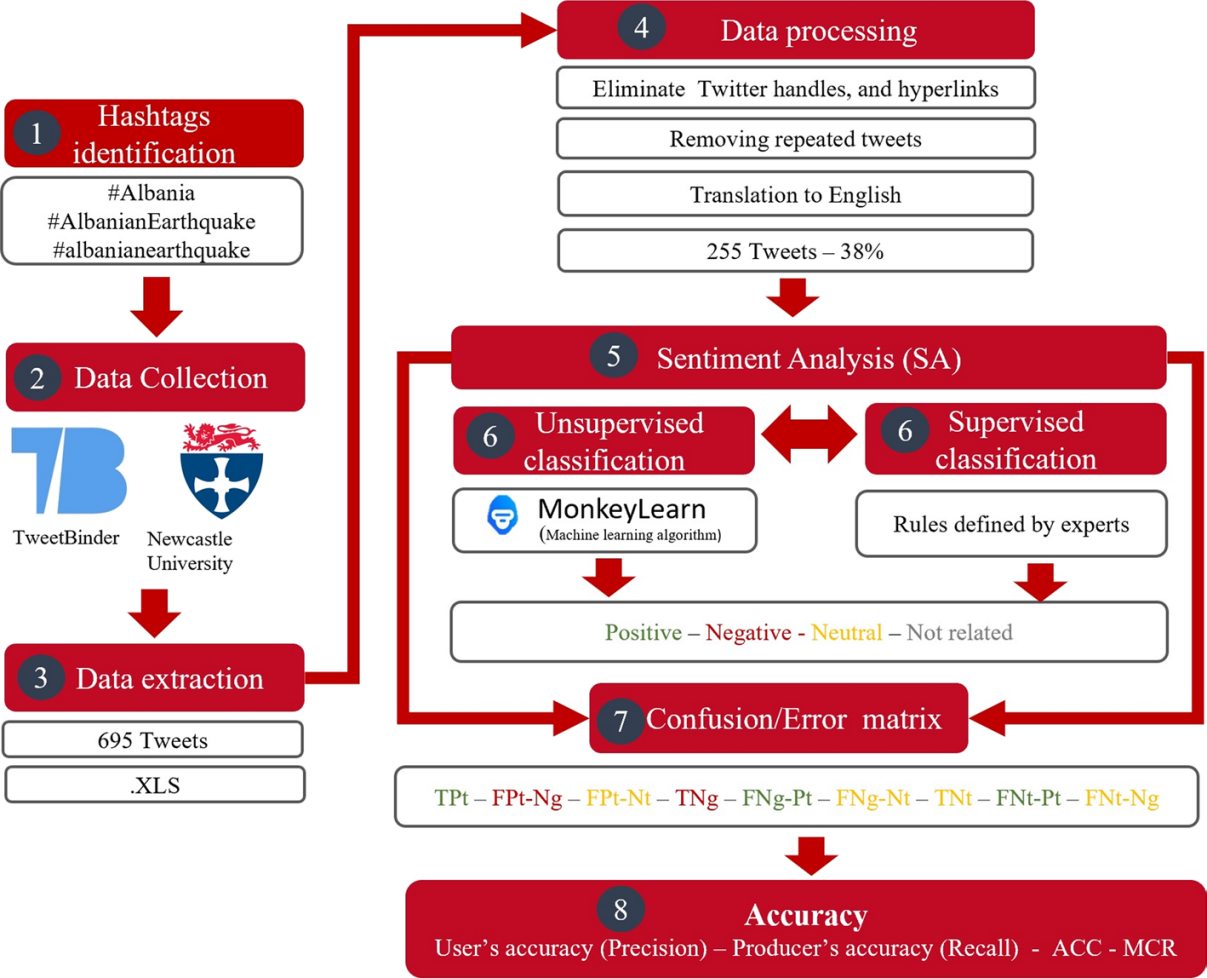
Methodology

The text data in the dataset of original tweets were also classified according to these rules and used as reference data to test the SA model's accuracy. The result of this supervised (rule-based) classification will be named:' truth'. The classification outcome from the pre-trained SA model was compared with the outcome from rule-based classification to determine the machine learnings platform's performance. The comparison of the classification outcomes is listed in Table 2. To test the performance of the unsupervised classification algorithm, we use a confusion matrix (a technique for measuring the accuracy of a classification algorithm). There are three polarities: positive, negative and neutral, of which one will be correct. At the same time, the other two will be incorrect, e.g. if a tweet is classified as positive by the pre-trained model and also classified positive by experts, the machine learning platform is correct: true positive (TPt). In contrast, if the tweet is classified as negative or neutral, then the classification is incorrect: false negative (FNg) or false neutral (FNt). Including the other two polarities (negative and neutral) for each tweet, there are nine possible permutations that we label with the correctness of the classification (first) and the actual category (second): incorrectly classified as positive when actually a negative polarity: FPtNg; incorrectly classified as positive when actually a neutral polarity: FPtNt, etc. The nine permutations, together with the number of tweets matching that permutation, are presented in Table 3. In addition to the confusion matrix, we also estimate the overall accuracy (ACC) and the misclassification rate (MCR) of the pre-trained model of SA developed by MonkeyLearn. The flow diagram of the methodology applied can be observed in Fig. 3, and the confusion matrix is available online (Contreras et al. 2021c) (see data availability statement).

**Table 2:** Comparison of the machine learning platform and rule-based polarity classification results

Polarity	Unsupervised classification algorithm-based		Supervised classification rule-based	
	Predicted	Percentage (%)	Truth	Percentage (%)
Positive	79	31	136	53
Negative	53	21	71	28
Neutral	123	48	48	19
Total	255	100	255	100

**Table 3:** Instances of polarity classes and MonkeyLearn confidence metric

Instances	Abbreviation	Number	Confidence (average)
True positive – correctly identified positive polarity	TPt	73	0.71
False positive – actually a negative polarity	FPtNg	5	0.64
False positive – actually a neutral polarity	FPtNt	1	0.50
True negative—correctly identified negative polarity	TNg	41	071
False negative—actually a positive polarity	FNgPt	11	0.60
False negative—actually a neutral polarity	FNgNt	1	0.84
True neutral—correctly identified neutral polarity	TNt	46	0.72
False neutral—actually a positive polarity	FNtPt	52	0.67
False neutral—actually a negative polarity	FNtNg	25	0.63
Total	255

## Results

**Fig. 4:**
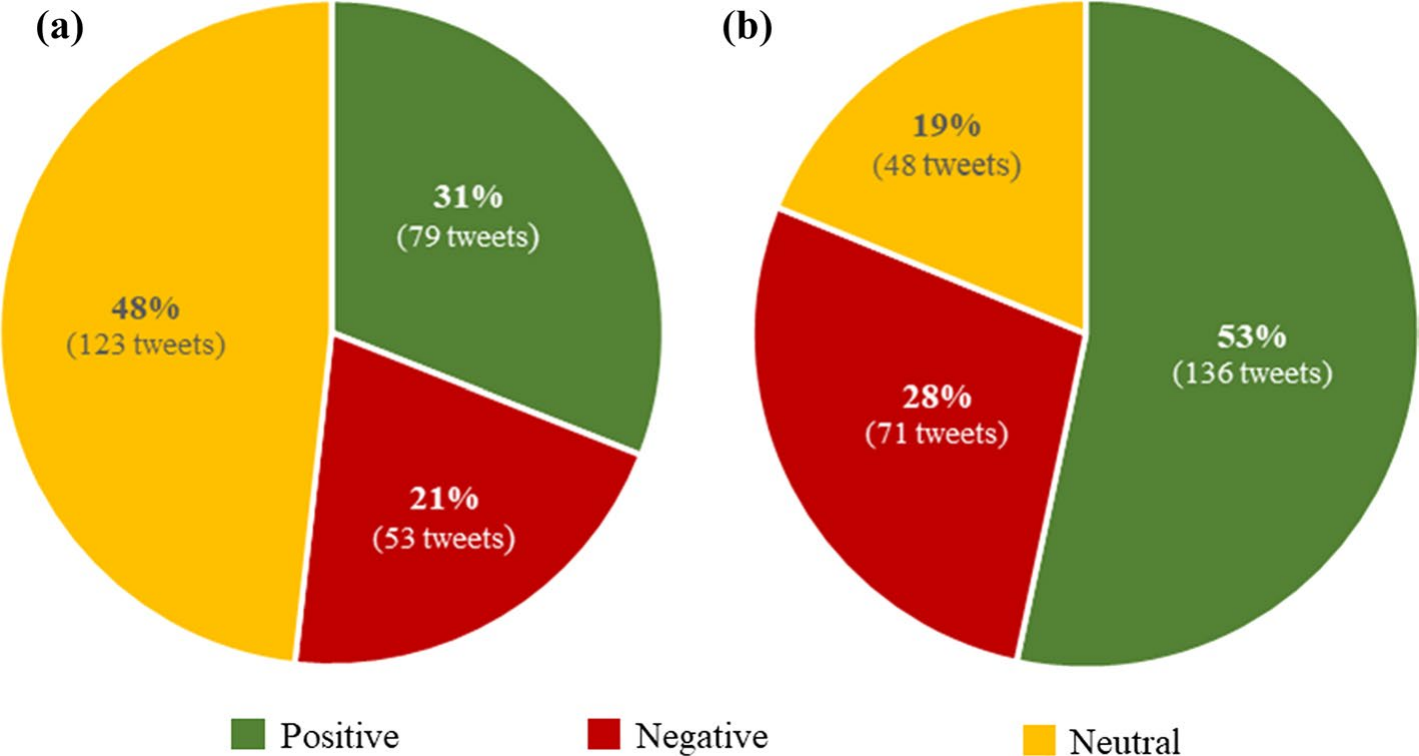
Polarity classification result. **a** Unsupervised classification (algorithm-based). **b** Supervised classification (rule-based)

The comparisons of the general results of the algorithm and rule-based polarity classification are listed in Table 2 and Fig. 4. Table 2 shows in the second column the proportion of supervised tweets that are classified as either positive, negative or neutral for comparison with the unsupervised classification (fourth column). Table 3 presents the number of correctly and incorrectly classified Tweets and the confidence metric produced by the MonkeyLearn algorithm.

To determine the accuracy of the unsupervised classification, we calculate the overall accuracy (ACC):

$${\text{ACC}} = {\text{TPt}} + {\text{TNg}} + {\text{TNt}}/\left( {{\text{TPt}} + {\text{TNg}} + {\text{TNt}} + {\text{FPt}} + {\text{FNg}} + {\text{FNt}}} \right)$$

$${\text{ACC}} = 73 + 41 + 46/\left( {73 + 41 + 46 + 6 + 12 + 77} \right)$$

$${\text{ACC}} = 160/255$$

$${\text{ACC}} = 0.63$$

While overall accuracy indicates how well the algorithm works, it does not indicate the accuracy of the classification per polarity. To test this, we have presented the discriminated results in a confusion matrix in Table 4, explained in the Discussion section. The diagonal numbers (bold coloured) in Table 4 indicate the tweets that were classified correctly, TPt, TNg, TNt (HSU 2016); while the other numbers represent misclassifications of the polarity, i.e. FPt ($\sum \mathrm{FPtNg}+\mathrm{FPtNt})$, FNg ($\sum \mathrm{FNgPt}+$ FNg-Nt) and FNt $(\sum \mathrm{FNtPt}+\mathrm{ FNtNg})$. The precision (i.e. the proportion of tweets of a particular polarity classified with that polarity) is calculated by taking the total number of correct classifications for each polarity and dividing it by the row's total (HSU 2016). The recall (i.e. the proportion of tweets assigned a particular polarity that is correctly classified) is calculated by taking the correct classification number for each polarity and divided by the column total.

**Table 4:** Confusion matrix testing the accuracy of the unsupervised classification

	Reference data					
	Polarity	Positive	Negative	Neutral	Classification overall	User's accuracy (precision)
Classified data	Positive	73	11	52	136	0.54
	Negative	5	41	25	71	0.58
	Neutral	1	1	46	48	0.96
	Truth overall	79	53	82	255	
	Producer’s accuracy (recall)	0.92	0.77	0.37		

## Discussion

This research aims to test the accuracy of a pre-trained SA classification model. This model is based on the no-code machine learning platform: MonkeyLearn. We selected this platform among other no code machine learning tools (Wolff, 2020) because it is the most user-friendly and therefore a good choice to explore the feasibility of automating the classification process to extract meaningful information from text data obtained from SM, accessible for emergency response practitioners to make data-driven decisions. If we were only focused on assessing damage in buildings, then we would need the location of the Twitter users (no longer available) to map the spatial distribution of different damage states. However, suppose that were the aim of this research, we could still use pictures included in the tweets and determine their location using tools such as ‘Google images’ and ‘Google street view’ or contact the photographers or local researchers to georeference those pictures. This procedure was followed to produce a map with the damage location for the 2020 Zagreb earthquake in Croatia (Contreras et al. 2021a, b). Another option would have been to use the dataset of georeferenced comments collected through the LastQuake app’ (Bossu et al. 2018a, b, ) developed by the European Mediterranean Seismological Centre (EMSC).

The total number of tweets related to his case study is very low compared to the tweets collected for the cases of the 2020 Zagreb Earthquake (59,246) (Contreras et al. 2021a) and the 2020 Aegean earthquake (618,145) (Aktas et al. 2021). We believe (but not yet proven) that the number of tweets related to an emergency generated by an earthquake could depend on the magnitude, the intensity, the impact, the number of Twitter users in the affected zone, the observation period or the hashtags selected. We will test these hypotheses in further research.

It is important to clarify that sentiment words and phrases are essential but not enough for an accurate SA. Positive or negative sentiment words could have opposite polarities in different contexts. A sentence containing sentiment words may not express any sentiment. A sarcastic sentence with or without sentiment words or sentences without sentiment words are challenging to classify into a positive or negative polarity (Liu 2015). These problems explain why we do not have a simple binary classification (positive/negative). We have used a confusion matrix to test the accuracy of the pre-trained SA model over a confusion matrix as the former considers all nine instances of polarity rather than the usual four categories, (i.e. TPt, FPt, TNg, FNg).

There is a difference in the classification outcomes between the algorithm and the rule-based classification. SA's accuracy references the agreement rate between the outcome of automatic classification models based on algorithms and human evaluations (Maksimava 2020). However, research studies demonstrated that human raters only agree with each other between 65 and 80% of the time. The sentiment is regularly subjective; therefore, it is difficult to measure its accuracy. On average, researchers agree that SA needs to have at least 50% accuracy to be considered effective, while around 65% is considered good (Maksimava 2020). In this research, we observed an ACC of 63% with a corresponding misclassification rate of 37%, which we argue is acceptable for initial assessments.

Looking at Table 4, we can see that the producer's accuracy (which measures how good the algorithm is at correctly classifying tweets) is very good at assigning positive polarity (92%) good for negative tweets (77%) and poor for neutral tweets (37%). On the other hand, the user's accuracy is very good for neutral tweets (96%) and above 50% for positive and negative Tweets. The high user's accuracy for neutral tweets and poor producer's accuracy is because the machine learning platform overclassifies neutral tweets. This means that someone looking at a tweet classified by the machine learning platform has more chance of obtaining a neutral classification for a tweet than if it had been manually classified. This overestimation of neutral tweets comes from a difference between how the authors' have looked at the tweet and how the machine learning platform has learnt to classify the tweets. We are able to look at a tweet that has no emotive language but states something positive (such as a simple statement quoting a donation or shelter being provided, e.g. 'Merkel assured Rama that Germany will participate in the Donors Conference for Albania, on February 17. EU news Albanian earthquake') and infer positivity, whereas the algorithm cannot replicate this.

While these numbers are encouraging, it is important to try and understand why tweets may have been misclassified and which tweets were classified most reliably. Reliable tweets will aid decision-making, whereas responding to erroneous tweets may hinder relief efforts. In the classification performed by the machine learning platform, most of the tweets are classified as neutral, followed by positive, and finally, negative. According to the authors' rules, most tweets have a positive polarity, followed by negative and neutral. Tweets classified as FPtNg, FPtNt, FNgPt, FNgNt, FNtPt, and FNtNg most times show lower average confidence in the classification (0.67-0.50) than those classified as TPt, TNg and TNt (0.71–0.72).

One possible reason for the high positive polarity could be related to the Independence day of Albania. Seven tweets (3%) included explicit references to the Independence day of Albania, and while this is a small proportion, the pride in the population celebrating their independence could explain the high percentage of positive polarity in the text data, despite the earthquake. In this paper, we assess the accuracy of tweets, so we have only considered original tweets. To quantify an assessment of how positive or negative the sentiments of the community is regarding the emergency response and post-disaster recovery assessment, it is necessary to consider all tweets as this is a better reflection of the sentiment of the entire community (i.e. include retweets in the analysis) however this is beyond the scope of this paper.

The most reliable positive tweets were mainly related to the immediate response. Tweets thanking, solidarity, encouraging and condolence messages, recovery wishes, and references to the Albanian Independence Day were identified as TPt. Tweets about international support for mapping damages activated through the European Union (EU) Civil Protection Mechanism were classified as TPt. Tweets about search and rescue (SAR) actions undertaken from the Israel Defense Forces (IDF), Kosovo, Serbia, Macedonia, Montenegro, Greece, Italy and Romania and Los Angeles County Fire Department (LACOFD), the last one activated through the Office of Foreign Disaster Assistance (OFDA) by the United States Agency for International Development (USAID) were also classified as TPt. The increase in the number of volunteers and humanitarian action from Red Cross was identified as TPt. The collection of humanitarian aid by citizens and activities to collect funds and donations from citizens to support early recovery were also classified as TPt.

Reliable Tweets that expressed negative sentiments were related to the lack of accountability for casualties caused by the buildings that collapsed in Albania and tweets reporting injured people (both classified as TNg). Reports of casualties and injured people were also classified as TNg. Tweets commenting about impacts of aftershocks, destroyed infrastructure and bad construction practices were identified as TNg. Tweets reporting restrictions to inform about the impact of the earthquake were also classified as TNg. Tweets complaining about the lack of proper coverage of the impact of the Albanian earthquake 2019 by the press were identified as TNg. Problems with the management of assistance for the survivors were also identified as TNg. Expression of anxiety was classified as TNg. Tweets complaining about the low quality of the building damage survey undertaken after the earthquake on the 21 September 2019 (Bossu et al. 2020) and their consequences (two months later, one house collapsed, killing half of a family during the earthquake addressed in this paper) were equally classified as TNg.

For neutral polarity, Tweets reporting the cities affected by the earthquake and seismic information were identified as TNt. Tweets informing about geotechnical effects were also classified as TNt. Tweets containing information about technical specifications of items of humanitarian aid were classified as well as TNt. Tweets commenting about the characteristics of affected areas were identified as TNt. Tweets containing information about property insurance were additionally classified as TNt. Despite the careful selection of the hashtags, there are 18 (7%) unrelated tweets. The presence of unrelated tweets happens because some Twitter users included hashtags related to the earthquake to promote football games for example. The machine learning platform classified these unrelated tweets in the pre-trained SA model developed by MonkyeLearn as neutral, and we accepted as TNt.

According to the rules defined by the authors, five tweets with negative polarity were misclassified as positive (FPtNg). In three cases, this misclassification is explained because the analysis is done at the tweet level, and tweets mix sentences with different polarities. One case is a tweet written with sarcasm, which usually includes a negative polarity never recognised by algorithms because of the lack of context (Liu 2015; MonkeyLearn 2020a, b, c). The other tweet addressed an unrelated topic to the earthquake, however, it was included in the dataset because it contains the hashtags considered in the research. One tweet was misclassified as positive when it is neutral (FPtNt), according to the rules defined by authors because it discusses seismic aspects.

Also, according to the rules defined by the authors, 11 tweets were misclassified as negative when they are positive (FNgPt). Cristiano Ronaldo and Gianluigi Buffon, forward and goalkeeper from the Juventus football club at that time, met young survivors. At the same time, the American singer Bebe Rexha and the Kosovo-Albanian rapper Capital, Tweeted about the disaster. The visit from prominent people contributes to improving the mood among survivors, and their tweets to the awareness of the situation potentially could attract more humanitarian support. Our rules consider volunteer initiatives to be positive. Tweets contain text data related to early recovery activities and donations, and fundraising activities are also considered positive. We currently cannot explain why the algorithm misclassifies some of these tweets, e.g. 'While the death toll raised to 31, solidarity with those affected by the Albanian Earthquake has been huge. Tonight in Durrës nobody is going to sleep outside in tents after families and hotels in the area were able to accommodate hundreds of those in need'.

In the same way, we also classify expressions of condolence and solidarity as positive; however, the algorithm is less reliable as these often include expressions of sadness or sorrow. Another tweet containing seismic information was misclassified as negative when we classify as neutral (FNg-Nt), e.g. 'Previous internet detection in Albania 10 min ago is confirmed by seismic signal'. Fifty-two tweets were misclassified as neutral when they are positive (FNtPt). The delivery of humanitarian assistance and the presence of volunteers we classify as positive because they are oriented to meet the needs of the affected population. The Government visit to the SAR team is considered positive. Humanitarian actions such as sheltering, fundraising and donations we also classified as positive. This is due to the tweets being written in a matter-of-fact way and therefore are classified as neutral, e.g.:’I leave the house for shelter for our compatriots after that natural disaster. The house has an accommodation capacity for two families (10 people) and is located in the village of Medvec respectively 3 km away from Prishtina Airport. Contact No. + ***********. Albania.Albanian Earthquake’.

Twenty-five tweets were misclassified as neutral when they are negative (FNtNg). Damages in buildings were wrongly reported as neutral (which is an important issue for response and recovery). Others reflect the lack of preparedness in Albania to face the challenges imposed by the earthquake. Another tweet detected as neutral should have been classified as negative because it reports the lack of coherence from the government regarding the management of humanitarian aid. Other tweets reported the end of SAR efforts, and others contained xenophobic messages.

The precision metric indicates the probability that a polarity predicted by the algorithm is in agreement with the rule-based or supervised classification. This is referred to as reliability. In our sample of tweets related to the emergency and early recovery phase after the 2019 Albanian earthquake, the predicted polarity with the highest level of precision in its classification is the neutral polarity (96%), followed by the negative (58%) and the positive (54%). We consider these numbers acceptable for a no-code machine learning tool that has been trained with text data not related to earthquake reconnaissance. In further research, we will classify this data based on a customised model trained with the same text data to be classified and check if precision and accuracy in the classification increase. The recall, also known as sensitivity or probability of detection, indicates the probability that a specific polarity is correctly classified as it is. In this case, the polarity with the highest probability of detection is positive, followed by neutral and negative. These differences could be explained by using words that cannot be associated with a specific polarity. According to the ACC results, there is an acceptable probability that a tweet will be classified in the correct polarity using the predefined SA model developed by MonkeyLearn.

## Conclusions

In this paper, we have looked at the accuracy of an unsupervised classification algorithm to obtain polarities of tweeted text. We performed an unsupervised classification and compared the obtained polarities to those obtained using supervised classification. Considering the overall accuracy metric (ACC = 0.63) we can conclude that the pre-trained SA model developed by MonkeyLearn is acceptable for a quick estimation of the polarity of text data collected during the emergency and early recovery phase after an earthquake. The SA classification machine learning platform generally recognises positive and negative polarities, but neutral tweets are overclassified.

MonkeyLearn machine learning platform has a confidence metric indicating how confident the platform has made a correct classification. Taking the average of the confidence metric as a simple indicator of the confidence of the classification, the correctly classified Tweets (TPt, TNg and TNt) have a score above 0.7. In contrast, the misclassified tweets (FPtNg, FPtNt, FNgPt, FNgNt, FNtPt, and FNtNg) have a score between 0.5 and 0.67 (except for FNgNt which only had 1 tweet, which was probably misclassified as negative as it had the word 'seismic' in it). While the difference between correctly and incorrectly classified tweets is relatively small, it is consistent.

For a large number of tweets (which is the case for a disaster), the unsupervised classification will save a great deal of effort over supervised. We also argue that another advantage of using a pre-trained SA classification model based on a machine learning platform is that it has the potential to improve the consistency of the polarity classification of the entire dataset of text (except for sarcasm or colloquial expressions).

Looking at SM can provide a general background of the situation after the earthquake, related to seismic information (epicentre, depth, aftershocks), the number of injured and casualties, reports of damages in buildings, critical infrastructure (CI) and problems with construction practices, and geotechnical effects of the earthquake, preparedness level of the affected area, solidarity actions (donations and fundraising activities) and messages, SAR efforts, preliminary PDNA's, emergency management successes and failures, political aspects and prominent people, institutions and NGO's involved in the emergency response actions and early recovery initiatives.

We suggest that we could improve the classification accuracy by making the SA analysis at the sentence level rather than at the tweet level. A tweet can contain different sentences with different polarities. Most tweets are classified as neutral by the machine learning platform because they are not customised to detect positive and negative aspects that contribute to or diminish emergency response and early recovery efforts. This problem may be overcome if a topic classification was first performed (MonkeyLearn 2020a, b, c) or topic content analysis (Karami et al. 2020) (another NPL technique), and only those tweets that are exclusively related to these topics considered to make the automatic assessment. However, it would be interesting to test the performance of this algorithm with other datasets of tweets collected during the emergency and early recovery phases after the 22nd March earthquake in Zagreb (So et al. 2020; Contreras et al. 2021a) and the 30th October 2020 Aegean earthquake (Aktas et al. 2021). Shorter tweets are mainly correctly classified. This observation can be explained by the fact that fewer polarities are expressed in shorter tweets.

To deal with unrelated tweets, we are considering three strategies for other cases: (1) Avoid selecting hashtags, which include only the name of the affected area, e.g. #Albania, (2) Eliminate the unrelated tweets from the dataset, and or (3) customise a SA model including the category of 'unrelated'. The usefulness of tweets is very variable. So, we believe there is a case for training and empowering citizens as sensors (Cervone and Hultquist 2018; Fallou et al. 2020) to safely contribute to the emergency response with meaningful comments, tweets, and posts on SM. These contributions should include intensity reports (Bossu et al. 2018b, a), the location of damaged buildings, injured and casualties, needs and damages, failures in lifelines, and images of damages in buildings with enough background to make easy the georeferencing of the damages (Bossu et al. 2020; Contreras et al.2021a, b, c, d, e, f, g).

## Data Availability

The complete error matrix is available online at: https://doi.org/10.25405/data.ncl.14687163.v1. Dataset.citation: Contreras Mojica, Diana; Wilkinson, Sean; Alterman, Evangeline (2021): Supervised & unsupervised polarity classification of Twitter data related to the Albania 2019 earthquake. Newcastle University. Dataset. https://doi.org/10.25405/data.ncl.14604654.v1

## Code availability

We use the no-code AI platform: MonkeyLearn to undertake the unsupervised classification.
